# Fine Tuning of Defects Enables High Carrier Mobility and Enhanced Thermoelectric Performance of n-Type PbTe

**DOI:** 10.1021/acs.chemmater.2c03542

**Published:** 2023-01-09

**Authors:** Siqi Wang, Cheng Chang, Shulin Bai, Bingchao Qin, Yingcai Zhu, Shaoping Zhan, Junqing Zheng, Shuwei Tang, Li-Dong Zhao

**Affiliations:** † School of Materials Science and Engineering, Beihang University, Beijing100191, China; ‡ Institute of Science and Technology Austria, Am Campus 1, 3400Klosterneuburg, Austria; § School of Materials Science and Engineering, Liaoning Technical University, Fuxin123000, China

## Abstract

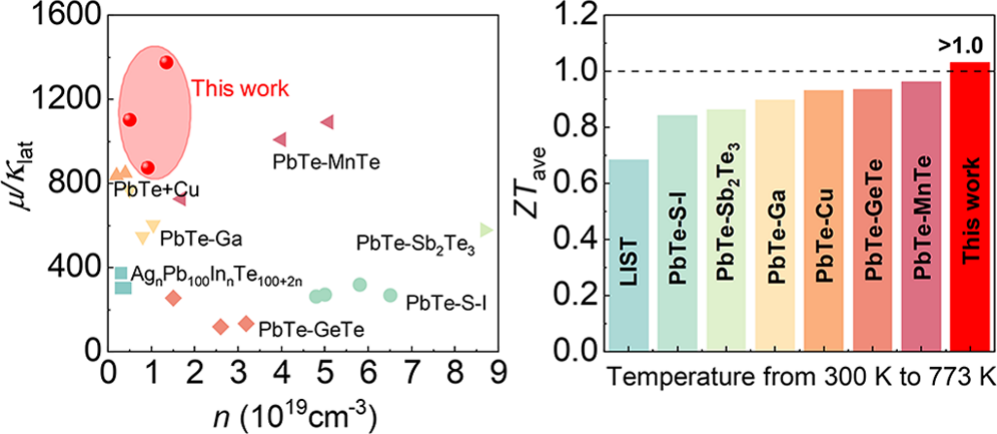

High carrier mobility is critical to improving thermoelectric performance over a broad temperature range. However, traditional doping inevitably deteriorates carrier mobility. Herein, we develop a strategy for fine tuning of defects to improve carrier mobility. To begin, n-type PbTe is created by compensating for the intrinsic Pb vacancy in bare PbTe. Excess Pb^2+^ reduces vacancy scattering, resulting in a high carrier mobility of ∼3400 cm^2^ V^–1^ s^–1^. Then, excess Ag is introduced to compensate for the remaining intrinsic Pb vacancies. We find that excess Ag exhibits a dynamic doping process with increasing temperatures, increasing both the carrier concentration and carrier mobility throughout a wide temperature range; specifically, an ultrahigh carrier mobility ∼7300 cm^2^ V^–1^ s^–1^ is obtained for Pb_1.01_Te + 0.002Ag at 300 K. Moreover, the dynamic doping-induced high carrier concentration suppresses the bipolar thermal conductivity at high temperatures. The final step is using iodine to optimize the carrier concentration to ∼10^19^ cm^–3^. Ultimately, a maximum *ZT* value of ∼1.5 and a large average *ZT*_ave_ value of ∼1.0 at 300–773 K are obtained for Pb_1.01_Te_0.998_I_0.002_ + 0.002Ag. These findings demonstrate that fine tuning of defects with <0.5% impurities can remarkably enhance carrier mobility and improve thermoelectric performance.

## Introduction

Thermoelectric materials can effectively utilize low-grade heat sources to generate electricity, such as industrial waste heat, automobile exhaust, solar energy, geothermal heat, *etc*.^1−4^ To date, the priority of thermoelectric research is to improve the conversion efficiency for more widespread applications. The conversion efficiency depends on the dimensionless figure of merit *ZT*, *ZT* = *S*^2^σ*T*/κ, where *S* is the Seebeck coefficient, σ is the electrical conductivity, *T* is the absolute temperature in kelvin, and κ is the total thermal conductivity (κ = κ_lat_ + κ_ele_ + κ_bi_, where κ_lat_ is the lattice thermal conductivity, κ_ele_ is the electronic thermal conductivity, κ_bi_ is the bipolar thermal conductivity). In general, thermoelectric enhancement relies heavily on a large Seebeck coefficient, high electrical conductivity, and low thermal conductivity. Nevertheless, the above three thermoelectric parameters are governed primarily by the carrier concentration and are not independently controllable, making them difficult to be optimized simultaneously.^5−9^ Researchers have conducted in-depth and fruitful research in upgrading thermoelectric performance *via* combined optimization of electrical and thermal transport. For electrical properties, optimizing carrier concentration,^10−12^ increasing carrier mobility,^13,14^ and modulating band structure^15−19^ are generally applied. For thermal properties, the traditional method is declining the lattice thermal conductivity *via* hierarchical structures, involving atomic-scale defects,^20−22^ nanoscale defects,^23−25^ and microscale defects.^26,27^

Among a variety of thermoelectric materials, PbTe is a representative thermoelectric material servicing at medium temperature. Numerous research studies have shown that *p*-type PbTe materials have achieved extraordinary thermoelectric properties. Many prominent *p*-type PbTe systems have realized *ZT* > 2.0, such as Pb_0.98_Na_0.02_Te,^28^ Pb_0.98_Na_0.02_Te–8%SrTe,^29^ Pb_0.98_Na_0.02_Te–6%MgTe,^30^ Pb_0.95_Na_0.05_Te–0.5%AgInSe_2_,^31^ and Pb_0.075_K_0.025_Te_0.7_S_0.3._^32^ In contrast, their counterpart, n-type PbTe, shows lower *ZT* values because of the large energy offset (∼0.45 eV) between the conduction bands, so further improvement of n-type PbTe is needed to match p-type PbTe. With time, we discovered several optimization methods that could bring about considerable intensification in the thermoelectric performance of n-type PbTe, such as doping and band engineering.^33^ However, even though traditional doping undoubtedly plays a significant role in increasing the carrier concentration, dopants inevitably reduce carrier mobility.^34−36^ Band engineering, as another attractive and feasible method, facilitates electrical performance by boosting the effective mass, while it is also detrimental to the carrier mobility.^37,38^ In general, these methods are capable of enhancing the maximum *ZT*, whereas a major drawback is their tendency to decrease carrier mobility, which limits the *ZT*_ave_.^39^ To ensure good thermoelectric properties over a wide temperature range, comparable n-type PbTe systems with high carrier mobility must be developed.

In this work, fine tuning of defects in PbTe is successively carried out to improve the carrier mobility. First, a small amount of excess Pb (0.004–0.012) is introduced into PbTe. As Pb enters the intrinsic Pb vacancies, n-type PbTe with relatively low carrier concentration is obtained. The dwindling vacancy defects improve carrier mobility, and the maximum carrier mobility reaches ∼3400 cm^2^ V^–1^ s^–1^ at 300 K in Pb_1.01_Te. Benefiting from the high carrier mobility, a maximum power factor of ∼31.3 μW cm^–1^ K^–2^ at 300 K is achieved in Pb_1.01_Te. Second, excess Ag is subsequently introduced to fill the remaining Pb vacancies. Results reveal that the comparatively low room-temperature carrier concentration of ∼5.5 × 10^17^ cm^–3^ leads to a high carrier mobility of ∼7300 cm^2^ V^–1^ s^–1^, resulting in a further increased power factor of ∼39.3 μW cm^–1^ K^–2^ at 300 K for Pb_1.01_Te + 0.002Ag. In particular, Ag atoms in the Pb_1.01_Te system undergo a dynamic doping process with increasing temperatures, first compensating the Pb vacancies and then entering more interstitial positions due to the temperature-dependent solubility of Ag in the PbTe matrix,^40^ which improves the carrier concentration from ∼5.5 × 10^17^ cm^–3^ at 300 K to ∼8.5 × 10^18^ cm^–3^ at 873 K for Pb_1.01_Te + 0.002Ag. Meanwhile, this unique dynamic doping behavior can effectively decouple the carrier concentration and carrier mobility across the entire temperature range and suppress bipolar diffusion at high temperatures. Last, 0.2% of I element (0.002I) is adopted to optimize the carrier concentration while maintaining superior carrier mobility than other n-type PbTe systems with similar carrier concentrations. Meanwhile, the minimum lattice thermal conductivity can reach as low as ∼0.5 W m^–1^ K^–1^ for Pb_1.01_Te_0.998_I_0.002_ + 0.002Ag. As a result, a maximum *ZT* of ∼1.5 at 773 K and a high average *ZT*_ave_ of ∼1.0 at 300–773 K are obtained for Pb_1.01_Te_0.998_I_0.002_ + 0.002Ag.

## Experimental Section

All raw materials used in the experiment were simple substances with more than 99.99% purity, including Pb, Te, Ag, and I. First, they were placed in quartz tubes according to the stoichiometric ratio. The quartz tubes were sealed below 10^–4^ Pa and put into a furnace (slowly heating from room temperature to 1323 K for 24 h, holding for 10 h, and finally cooling to room temperature). After the temperature program finished, some silver ingots were obtained, which were then ground into powder for proceeding spark plasma sintering (SPS-211Lx, Dr. Sinter). Finally, the sintered cylindrical, small pieces were cut into rectangles of 10 mm × 3 mm × 3 mm and slices of 8 mm × 8 mm × 1.5 mm to perform electrical and thermal performance tests. The electrical performance was measured by Cryoall CTA (electrical conductivity, σ and Seebeck coefficient, *S*) and Lake Shore 8400 Series (carrier density, *n*) instruments. The thermal performance was tested by a Netzsch LFA 457 (thermal diffusivity, *D*). The thermal conductivity, κ, was estimated by κ = *D* × *C*_p_ × ρ, where *C*_p_ is the specific heat capacity based on Debye’s law and ρ is the sample density calculated by the ratio of mass to volume. The optical band gap was measured by a Fourier transform infrared spectrometer, IRAffinity-1S, based on the infrared diffuse reflection method.

## Results and Discussion

In this study, the method of fine tuning of defects is used in the PbTe system to achieve superior electrical characteristics while maintaining a relatively low thermal conductivity. Supporting Information to create n-type PbTe, a modest quantity of Pb was first introduced, filling the intrinsic Pb vacancies, which increases carrier mobility and lowers thermal conductivity. Second, excess Ag is adopted for Pb_1.01_Te to fill the remaining Pb vacancies and also occupy interstitial sites, allowing the Pb_1.01_Te + *x*Ag system to achieve higher carrier concentration, ultrahigh carrier mobility, and lower thermal conductivity simultaneously across the entire temperature range. Third, the Iodine element is adopted to enhance the carrier concentration, further improving the power factor and restraining the lattice thermal conductivity in the whole temperature range.

### Thermoelectric Transport Performance of Pb_1+*x*_Te

Figure S1a shows the powder X-ray diffraction (XRD) patterns of Pb_1+*x*_Te (*x* = 0, 0.004, 0.006, 0.008, 0.01, 0.012), and the corresponding lattice parameters are given in Figure S1b. All Pb_1+*x*_Te samples are NaCl-type cubic structures without extra diffraction peaks detected, and the lattice parameters increase slightly with excess Pb.

**Figure 1:**
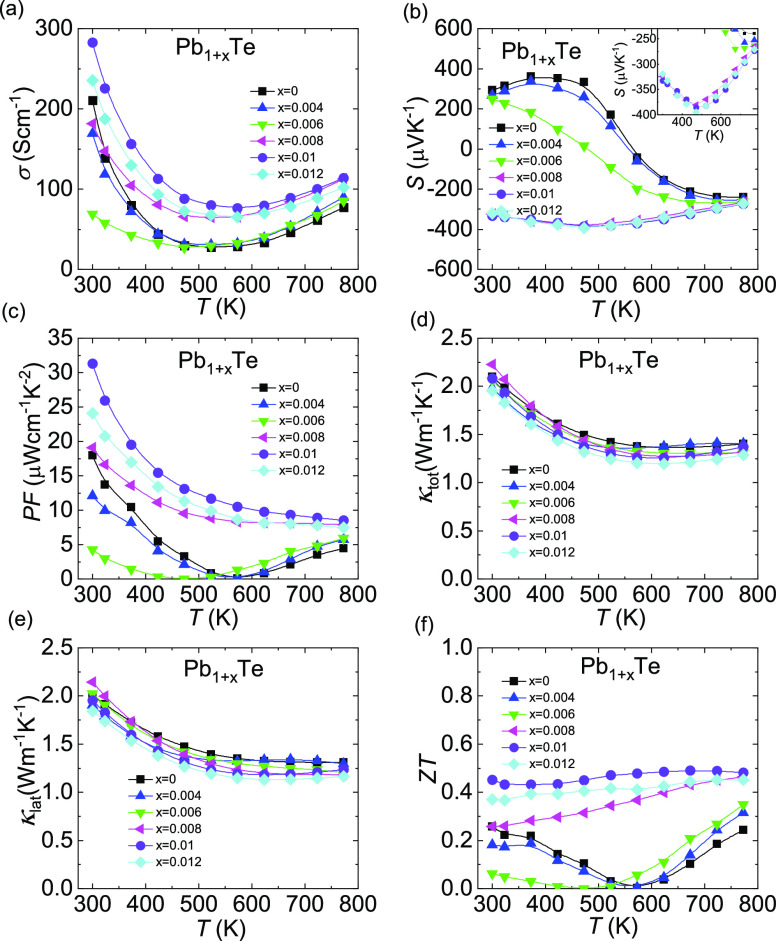
Thermoelectric transport performance of Pb_1+*x*_Te (*x* = 0, 0.004, 0.006, 0.008, 0.01, 0.012): (a) electrical conductivity, (b) Seebeck coefficient, (c) power factor, (d) total thermal conductivity, (e) lattice thermal conductivity, and (f) *ZT* value.

As can be seen in Figure 1a, the electrical conductivities decrease first and then increase with increasing Pb amount. Correspondingly, Pb_1+*x*_Te undergoes a p–n transition at low temperatures, as shown in Figure 1b. Specifically, the Seebeck coefficient of bared PbTe is positive at low temperatures; however, when *x* ≥ 0.008, the Seebeck coefficient is completely negative, resulting in n-type PbTe throughout the measuring temperature range. The combined behavior of electrical conductivity and Seebeck coefficient indicates that the hole concentration in PbTe is suppressed by excess Pb. In Figure 1c, benefitting from the improved electrical conductivity and Seebeck coefficient, the power factor is enhanced significantly, and the maximum value, i.e., room-temperature value, increases notably from ∼18.0 μW cm^–1^ K^–2^ for PbTe to ∼31.3 μW cm^–1^ K^–2^ for Pb_1.01_Te. In Figure 1d, the total thermal conductivity of PbTe reduces slightly with excess Pb, and the room-temperature value decreases from ∼2.1 to ∼2.0 Wm^–1^ K^–1^ caused by diminished lattice thermal conductivity, as shown in Figure 1e. Pb_1+*x*_Te shows higher electronic thermal conductivity, as shown in Figure S2b, because excess Pb fills the intrinsic Pb vacancies, thereby creating weaker carrier scattering. Ultimately, an average *ZT* value of ∼0.5 for Pb_1.01_Te is obtained throughout the whole measuring temperature range, as shown in Figure 1f.

**Figure 2:**
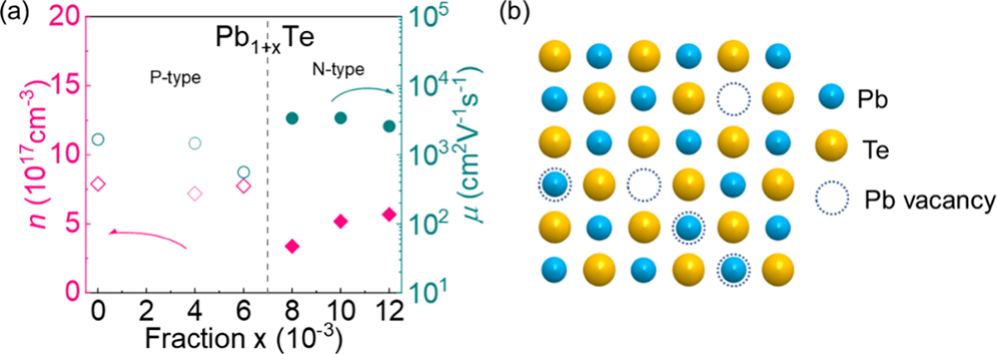
(a) Room-temperature carrier concentration and carrier mobility as a function of Pb and (b) atomic schematic diagram of Pb_1+*x*_Te.

To understand the electrical transport evolution of Pb_1+*x*_Te, we carried out the Hall measurement. In Figure 2a, the carrier concentration of ∼10^17^ cm^–3^ in the n-type Pb_1+*x*_Te system is relatively low, not as outstanding as the common doping approaches, such as PbTe–Sb^41^ (∼5.2 × 10^19^ cm^–3^), PbTe–I^42^ (∼1.8 × 10^19^ cm^–3^), PbTe–Bi^43^ (∼2.0 × 10^19^ cm^–3^), *etc*. With the increasing Pb fraction, the carrier concentration is gradually elevated, indicating that more and more Pb enters Pb vacancies. Based on the inverse relationship between carrier concentration and carrier mobility, relatively low carrier concentration brings about high carrier mobility since reduced Pb vacancies alleviate the charge carrier scattering, and the maximum carrier mobility can reach ∼3400 cm^2^ V^–1^ s^–1^ for Pb_1.01_Te. Figure 2b shows the atomic schematic diagram of the Pb_1+*x*_Te system, graphically depicting the phenomenon that excess Pb atoms occupy the intrinsic Pb vacancies.

### Electrical Transport Performance of Pb_1.01_Te + *x*Ag

The powder X-ray diffraction (XRD) measurements of Pb_1.01_Te + *x*Ag (*x* = 0–0.005) samples are performed, as shown in Figure S4a. All samples are NaCl cubic structures, and no peaks of impurities are found. Likewise, the small amount of excess Ag results in a slight expansion of the lattice parameters, as shown in Figure S4b.

**Figure 3:**
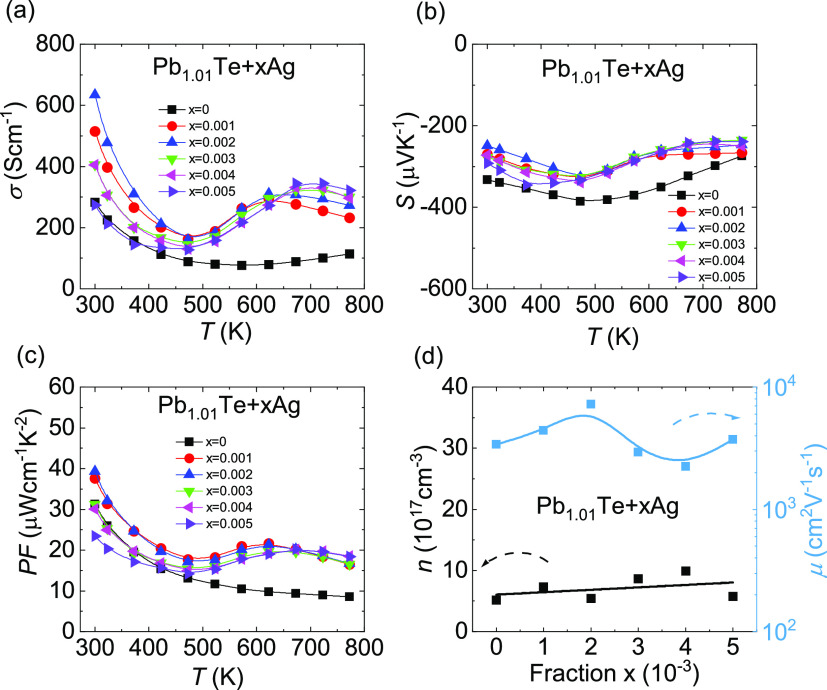
Electrical transport performance of Pb_1.01_Te + *x*Ag (*x* = 0–0.005): (a) electrical conductivity, (b) Seebeck coefficient, (c) power factor, and (d) room-temperature carrier concentration and carrier mobility as a function of Ag.

Figure 3 shows that excess Ag significantly improves the electrical transport performance of the Pb_1.01_Te + *x*Ag system. Figure 3a shows a clear increase in electrical conductivity throughout the whole temperature range. In particular, the electrical conductivity increases anomaly above 500 K. In Figure 3b, the negative Seebeck coefficients prove that Pb_1.01_Te + *x*Ag is electron-dominated n-type material. The introduction of Ag reduces the Seebeck coefficient, and the maximum absolute value decreases from ∼385.9 μV K^–1^ for Pb_1.01_Te to ∼249.0 μV K^–1^ for Pb_1.01_Te + 0.002Ag. The Pisarenko curve in Figure S5 reveals that the effective mass of the Pb_1.01_Te + *x*Ag system remains approximately 0.22m_0_, indicating that the depressed Seebeck coefficient of Pb_1.01_Te + *x*Ag is attributed to the elevated carrier concentration rather than the reduced effective mass. As a result, significantly incremental electrical conductivity and abated Seebeck coefficient further enhance the power factor compared to Pb_1.01_Te. In particular, a superior room-temperature power factor of ∼39.3 μW cm^–1^ K^–2^ is acquired for Pb_1.01_Te + 0.002Ag, as shown in Figure 3c. Also, the power factor above 500 K is twice higher as that of Pb_1.01_Te. Figure 3d shows the carrier concentration and carrier mobility of Pb_1.01_Te + *x*Ag at room temperature. Carrier concentration increases as the Ag fraction rises; however, carrier mobility is not impaired. Particularly, the maximum carrier mobility is increased by more than twice from ∼3400 cm^2^ V^–1^ s^–1^ for Pb_1.01_Te to ∼7300 cm^2^ V^–1^ s^–1^ for Pb_1.01_Te + 0.002Ag.

**Figure 4:**
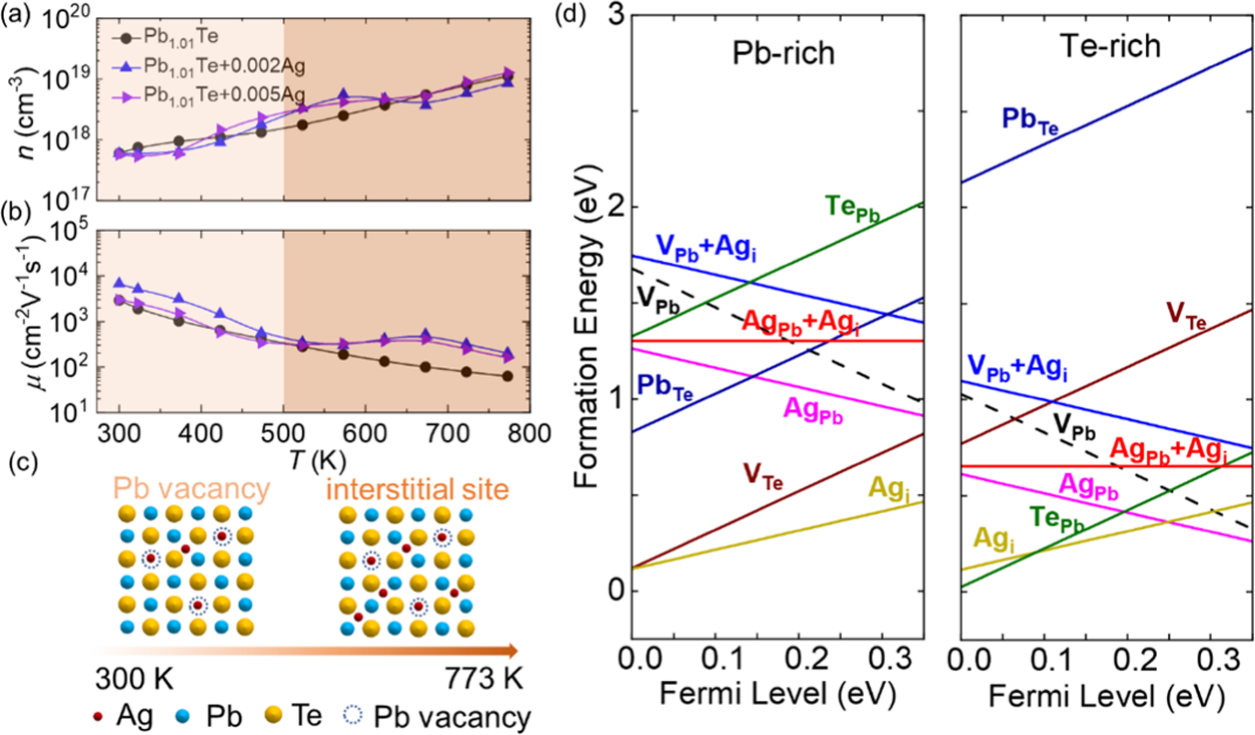
Dynamic doping process in Pb_1.01_Te + *x*Ag: (a) carrier concentration and (b) carrier mobility as a function of temperature, (c) atomic schematic diagram, and (d) calculated defect formation energies of PbTe.

To get an insight into the remarkable electrical transport properties of Pb_1.01_Te + *x*Ag, we measured the temperature-dependent carrier concentration, as shown in Figure 4a,b. The carrier concentrations of bare PbTe and Pb_1.01_Te increase with increasing temperatures, as shown in Figure S6, which is derived from the low carrier concentration and thus pronounced bipolar effect at high temperatures.^44^ However, Pb_1.01_Te + *x*Ag systems still exhibit increasing carrier concentration throughout the temperature range even though their bipolar effect is suppressed (the suppressed bipolar effect is evidenced by the low bipolar thermal conductivity, which is discussed in detail in the next section). We attribute the abnormal temperature-dependent behavior of carrier concentration to Ag-induced dynamic doping.^40^ The dynamic doping process in Pb_1.01_Te + *x*Ag is initiated by the multiple occupancies of Ag atoms with increasing temperatures, and Figure 4c displays the phenomena in detail. At 300–500 K, Ag atoms compensate for intrinsic Pb vacancies and interstitials, enhancing carrier mobility, as shown in Figure 4b. Above 500 K, more Ag atoms occupy interstitial positions to provide excess electrons for the system, resulting in a higher carrier concentration than the Pb_1.01_Te sample. It is noteworthy that the carrier mobilities of Pb_1.01_Te + Ag at high temperatures are also higher than those of Pb_1.01_Te, which can be explained by the suppressed bipolar effect. As a result, the carrier mobility of Pb_1.01_Te + Ag is greater than that of Pb_1.01_Te in the entire temperature range.

To further reveal the role of Ag in PbTe, we utilized first principles to calculate various defects and their formation energy in the system, such as vacancies, interstitials, antisites, and Ag-filled intrinsic vacancies. Figure 4d depicts the calculated formation energy of related defects. Based on the existence of Pb vacancies, the formation energy of Pb vacancies in the Pb-rich system is greater than that in the Te-rich system, implying that Pb vacancies are more difficult to form in the Pb-rich condition. For Pb_1.01_Te (Pb-rich system), the formation energy of Ag_Pb_ (Ag-occupied Pb vacancies) is lower than that of V_Pb_ (Pb vacancies), indicating that the introduction of Ag reduces the Pb vacancies, thereby lowering the content of point defects and improving the carrier mobility. The formation energy of Ag_i_ (Ag interstitials) is also very low in both Pb-rich and Te-rich systems, demonstrating that Ag not only fills Pb vacancies but also occupies the interstitials in PbTe. The calculated results of defect formation energy well support the experimental results and verify the role of the Ag element in improving the electrical transport performance of the Pb_1.01_Te system.

### Thermal Transport Performance of Pb_1.01_Te + *x*Ag

**Figure 5:**
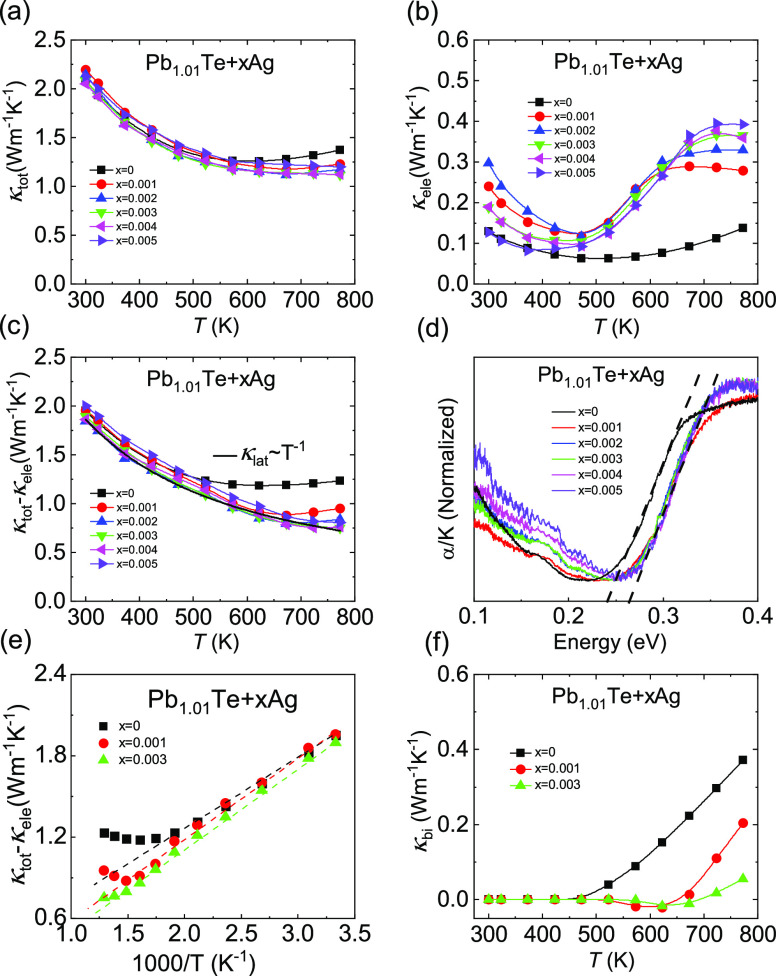
Thermal transport performance of Pb_1.01_Te + *x*Ag (*x* = 0–0.005): (a) total thermal conductivity, (b) electronic thermal conductivity, (c) κ_tot_ – κ_ele_ as a function of *T*, (d) room-temperature band gap, (e) κ_tot_ – κ_ele_ as a function of 1000/*T*, and (f) bipolar thermal conductivity.

Figure 5 displays the temperature-dependent thermal properties of Pb_1.01_Te + *x*Ag. In Figure 5a, the total thermal conductivity decreases slightly. The electronic thermal conductivity is calculated from equation κ_ele_ = *L*σ*T* (*L* is the Lorenz number, as shown in Figure S7a, determined by the single parabolic band (SPB) model). Because of the greater electrical conductivity, the Pb_1.01_Te + *x*Ag system displays much higher electronic thermal conductivity than Pb_1.01_Te, as shown in Figure 5b. Figure 5c presents the lattice thermal conductivity κ_lat_ calculated by κ_lat_ = κ_tot_ – κ_ele_ as a function of temperature. In the high-temperature region, all of the Pb_1.01_Te + *x*Ag samples exhibit lower κ_lat_ than Pb_1.01_Te since the elevated electronic thermal conductivity of Pb_1.01_Te + *x*Ag effectively suppresses the bipolar diffusion. Excess Ag depresses the minimum lattice thermal conductivity from ∼1.2 Wm^–1^ K^–1^ for Pb_1.01_Te to ∼0.7 Wm^–1^ K^–1^ for Pb_1.01_Te + 0.005Ag. The solid black line in Figure 5c is the theoretical lattice thermal conductivity of PbTe without bipolar thermal conductivity. The theoretical lattice thermal conductivity as a function of the temperature follows the relationship^30^
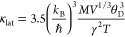
where *k*_B_ is the Boltzmann constant, *ℏ* is the Planck constant, *M* is the average atomic mass, *V* is the average atomic volume, θ_D_ is the Debye temperature, and γ is the Gruneisen constant. The κ_tot_ – κ_ele_ values of Pb_1.01_Te + *x*Ag samples coincide with the calculated values, indicating that the bipolar diffusion is suppressed in Pb_1.01_Te + *x*Ag. Meanwhile, the enlarged band gap is responsible for restrained bipolar diffusion due to the following formula^45,46^
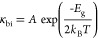
where *A* is the constant and *E*_g_ is the band gap. Ag enlarges the band gap in Figure 5d, thereby reducing the bipolar thermal conductivity. The (κ_tot_ – κ_ele_) – 1000 *T*^–1^ relationship is plotted in Figure 5e to examine the contribution of bipolar thermal conductivity, revealing that the true lattice thermal conductivity should be a straight line, while the κ_tot_ – κ_ele_ values in the high-temperature region are higher than the predicted values, indicating that bipolar diffusion occurs in this region. According to Figure 5f, bipolar thermal conductivity is the difference between the solid line and the dotted line and decreases as the amount of Ag increases.

### *ZT* Values of Pb_1.01_Te + *x*Ag

**Figure 6:**
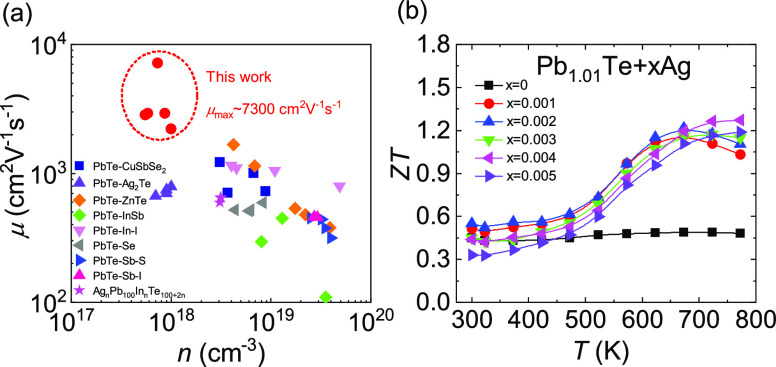
(a) Comparison of carrier mobility as a function of the carrier concentration of n-type PbTe and (b) *ZT* values of Pb_1.01_Te + *x*Ag.

Comparing room-temperature carrier mobility of the Pb_1.01_Te + *x*Ag system with those of the other n-type PbTe materials, we found that this system exhibits superior carrier mobility of ∼7300 cm^2^ V^–1^ s^–1^ at low carrier concentrations (∼10^17^ cm^–3^), as shown in Figure 6a, which is competitive in high-performance n-type PbTe-based materials. Finally, as a result of ultrahigh carrier mobility and suppressed bipolar thermal conductivity, room-temperature *ZT*, as shown in Figure 6b, is further enhanced from ∼0.5 for Pb_1.01_Te to ∼0.6 for Pb_1.01_Te + 0.002Ag, and high-temperature *ZT* is significantly increased from ∼0.5 for Pb_1.01_Te to ∼1.3 for Pb_1.01_Te + 0.004Ag.

### Thermoelectric Transport Performance of Pb_1.01_Te_1–*x*_I*_x_* + 0.002Ag

**Figure 7:**
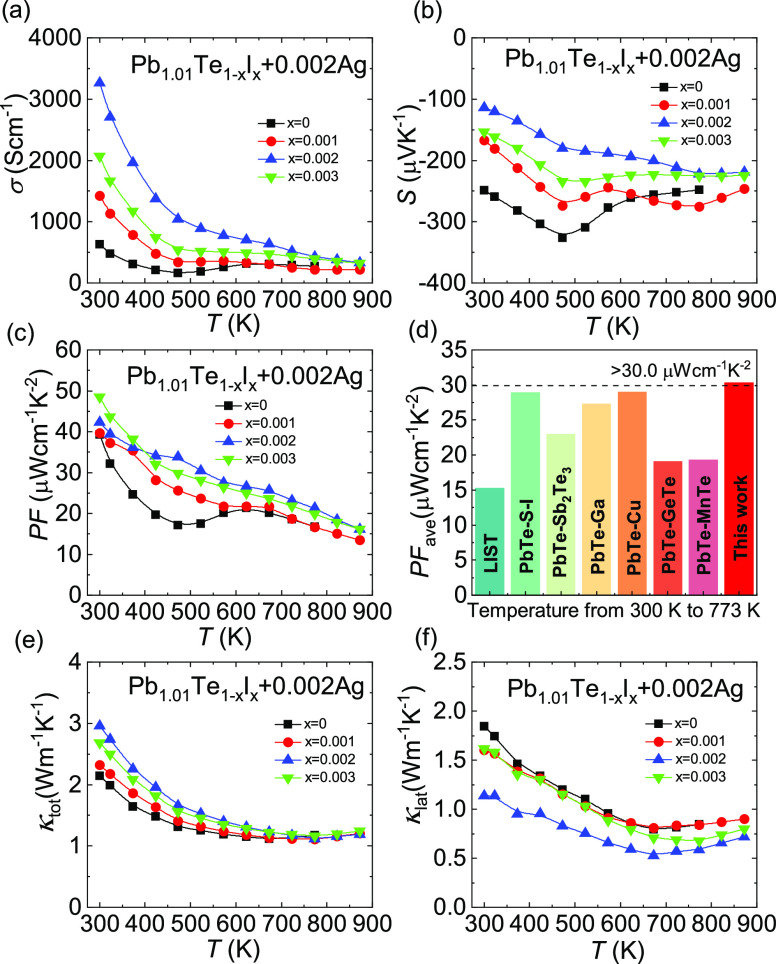
Thermoelectric transport performance of Pb_1.01_Te_1–*x*_I*_x_* + 0.002Ag (*x* = 0–0.003): (a) electrical conductivity, (b) Seebeck coefficient, (c) power factor, (d) average power factor, (e) total thermal conductivity, and (f) lattice thermal conductivity.

Based on Pb_1.01_Te + 0.002Ag with the maximum average *ZT*, I doping is employed to increase the carrier concentration, thereby improving the electrical performance. Figure S8 shows the phase identification of Pb_1.01_Te_1–*x*_I*_x_* + 0.002Ag (*x* = 0–0.003). No new peak is found in Figure S8a, and the samples still denote the NaCl structure. The lattice parameter decreases slightly in Pb_1.01_Te_1–*x*_I*_x_* + 0.002Ag, as shown in Figure S8b, because smaller I^–^ (∼2.06 Å) substitutes for larger Te^2–^ (∼2.11 Å). Figure 7 depicts the thermoelectric performance of Pb_1.01_Te_1–*x*_I*_x_* + 0.002Ag (*x* = 0–0.003). I doping can effectively improve the electrical conductivity, as shown in Figure 7a. The room-temperature electrical conductivity can be magnified from ∼633.5 S cm^–1^ for Pb_1.01_Te + 0.002Ag to ∼3267.8 S cm^–1^ for Pb_1.01_Te_0.998_I_0.002_ + 0.002Ag. The Seebeck coefficients of all samples in Figure 7b are negative, showing n-type semiconductor characteristics. The absolute value of the Seebeck coefficient of Pb_1.01_Te_1–*x*_I*_x_* + 0.002Ag decreases with increasing doping amount of the I element since the more I content, the higher the carrier concentration, as shown in Figure S9. The high electrical conductivity and low Seebeck coefficient of the Pb_1.01_Te_1–*x*_I*_x_* + 0.002Ag system indicate that the I element possesses extremely high doping efficiency in PbTe. The carrier concentration can be efficiently increased by doping with as little as 0.002I. Figure 7c shows that the power factor increases across the entire temperature range, with the maximum power factor increasing from ∼39.3 μW cm^–1^ K^–2^ for Pb_1.01_Te + 0.002Ag to ∼48.5 μW cm^–1^ K^–2^ for Pb_1.01_Te_0.998_I_0.002_ + 0.002Ag. The average power factor exceeds ∼30.0 μW cm^–1^ K^–2^ for Pb_1.01_Te_0.998_I_0.002_ + 0.002Ag, which is higher than those of other n-type PbTe systems (PbTe–S–I,^47^ PbTe–Sb_2_Te_3_–Sb–Cu_2_Te,^23^ Ag_n_Pb_100_InTe_100+2n_,^48^ PbTe + Cu,^49^ PbTe–GeTe,^50^ PbTe–MnTe,^51^ PbTe–Ga^52^), as shown in Figure 7d. In Figure 7e, the total thermal conductivity of Pb_1.01_Te_1–*x*_I*_x_* + 0.002Ag increases with increasing I content, primarily owing to an enhancement in electronic thermal conductivity. The lattice thermal conductivity decreases with increasing I content, as shown in Figure 7f, due to the enhanced phonon scattering. The minimum lattice thermal conductivity is reduced to ∼0.5 Wm^–1^ K^–1^ for Pb_1.01_Te_0.998_I_0.002_ + 0.002Ag.

**Figure 8:**
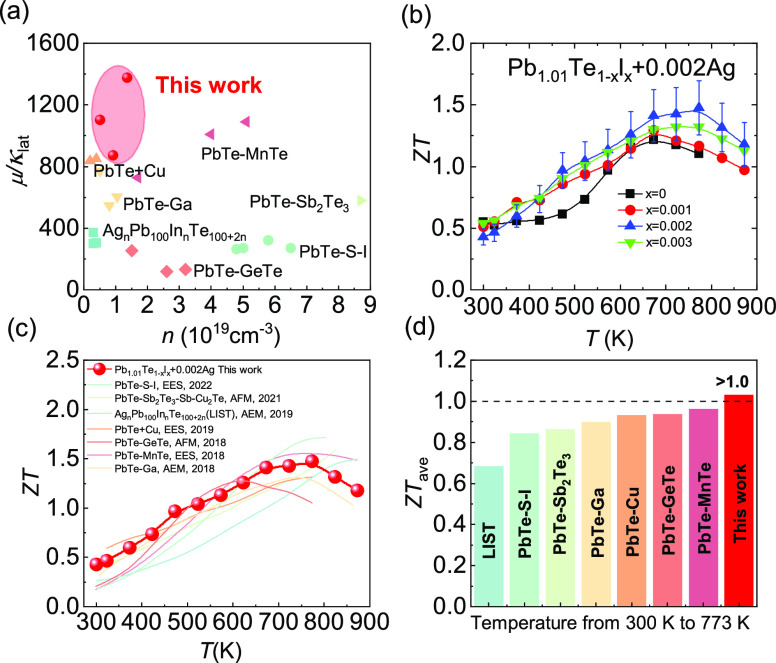
Comparisons of thermoelectric parameters of Pb_1.01_Te_1–*x*_I*_x_* + 0.002Ag with those of other systems: (a) diagram of μ/κ_lat_ and (b) *ZT* values of Pb_1.01_Te_1–*x*_I*_x_* + 0.002Ag. Comparisons of (c) *ZT* values and (d) average *ZT* values of Pb_1.01_Te_0.998_I_0.002_ + 0.002Ag with those of other PbTe systems.

To comprehensively evaluate the thermoelectric performance of the Pb_1.01_Te_1–*x*_I*_x_* + 0.002Ag system, the relationship between carrier concentration and the ratio of carrier mobility to lattice thermal conductivity (μ/κ_lat_) is plotted, as shown in Figure 8a. Compared with other high-performance n-type PbTe systems (PbTe–S–I,^47^ PbTe–Sb_2_Te_3_–Sb–Cu_2_Te,^23^ Ag_n_Pb_100_InTe_100+2n_,^48^ PbTe + Cu,^49^ PbTe–GeTe,^50^ PbTe–MnTe,^51^ PbTe–Ga^52^), the Pb_1.01_Te_1–*x*_I*_x_* + 0.002Ag system possesses higher μ/κ_lat_ values at lower carrier concentrations, indicating that a trace of Pb atoms (Pb-occupied Pb vacancies) and Ag atoms (Ag-occupied Pb vacancies) can effectively regulate the intrinsic defects in PbTe and reduce the scattering for charge carriers, protecting the charge carrier transport while scattering phonon. Accordingly, owing to a better balance between electrons and phonons, the *ZT* value increases in the whole temperature range, and the maximum *ZT* value is enhanced from ∼1.2 for Pb_1.01_Te + 0.002Ag to ∼1.5 for Pb_1.01_Te_0.998_I_0.002_ + 0.002Ag, as shown in Figure 8b. Figure 8c,d shows the comparison of *ZT* values and average *ZT*_ave_ values with various n-type PbTe materials, respectively. The large *ZT* values of Pb_1.01_Te_0.998_I_0.002_ + 0.002Ag in the whole temperature zone contribute to the excellent average *ZT*_ave_ value, reaching >1.0 at 300–773 K.

## Conclusions

This work has provided a deeper insight into designing fine-tuned defects to improve the carrier mobility of n-type PbTe. In particular, by introducing a small amount of Pb, the reduced Pb vacancy improves the carrier mobility to ∼3400 cm^2^ V^–1^ s^–1^ at 300 K for Pb_1.01_Te. Then, the room-temperature carrier mobility can reach as high as ∼7300 cm^2^ V^–1^ s^–1^ for Pb_1.01_Te + 0.002Ag due to Ag-induced dynamic doping. Finally, Iodine doping dramatically increases the carrier concentration while maintaining superior carrier mobility compared to other PbTe systems with only traditional dopants. The combination of high carrier mobility and the suppressed bipolar effect enables the significant enhancement of thermoelectric performance with a high *ZT* ∼1.5 at 773 K and average *ZT*_ave_ ∼1.0 at 300–773 K for n-type Pb_1.01_Te_0.998_I_0.002_ + 0.002Ag. These findings provide insights into balancing electron and phonon transport *via* fine tuning of defects, which could be a promising aspect of co-optimizing thermal and electrical performance of materials with instinct defects.

## Supporting Information Available

Density functional theory (DFT) calculations, formula of the average *PF*_ave_ value, phase identification of Pb_1+*x*_Te (*x* = 0, 0.004, 0.006, 0.008, 0.01, 0.0012) (Figure S1), thermoelectric transport properties as a function of temperature for Pb_1+*x*_Te (*x* = 0, 0.004, 0.006, 0.008, 0.01, 0.0012) (Figure S2), sample density of Pb_1+*x*_Te (*x* = 0, 0.004, 0.006, 0.008, 0.01, 0.0012) (Figure S3); phase identification of Pb_1.01_Te + *x*Ag (*x* = 0–0.005) (Figure S4), room-temperature Pisarenko plots of Pb_1.01_Te + *x*Ag (Figure S5), carrier concentration of PbTe and Pb_1.01_Te as a function of temperature (Figure S6), thermoelectric transport properties as a function of temperature for Pb_1.01_Te + *x*Ag (*x* = 0–0.005) (Figure S7), phase identification of Pb_1.01_Te_1–*x*_I*_x_* + 0.002Ag (*x* = 0–0.003) (Figure S8), room-temperature carrier density and carrier mobility as a function of Ag doping content (Figure S9), thermoelectric transport properties as a function of temperature for Pb_1.01_Te_1–*x*_I*_x_* + 0.002Ag (*x* = 0–0.003) (Figure S10), and sample density of Pb_1.01_Te_1–*x*_I*_x_* + 0.002Ag (*x* = 0–0.003) (Figure S11) (PDF)
