# Supplementary material for: Fine Tuning of Defects Enables High Carrier Mobility and Enhanced Thermoelectric Performance of n-Type PbTe
Source: Chem Mater. 2023 Jan 9;35(2):755–63. doi: 10.1021/acs.chemmater.2c03542 (PMC9878722; doi:10.1021/acs.chemmater.2c03542)
Supplement: Supplementary file 1 — cm2c03542_si_001.pdf [file cm2c03542_si_001.pdf]

Fine Tuning of Defects Enables High Carrier Mobility and Enhanced Thermoelectric Performance in *n*-type PbTe

Siqi Wang[1], *Cheng Chang*[2, *], *Shulin Bai*[3], *Bingchao Qin*[1], *Yingcai Zhu*[1], *Shaoping Zhan*[1], *Junqing Zheng*[1], *Shuwei Tang*[3], *Li-Dong Zhao*[1, *]

[1] School of Materials Science and Engineering, Beihang University, Beijing 100191, China.

[2] Institute of Science and Technology Austria, Am Campus 1, 3400 Klosterneuburg, Austria

[3] School of Materials Science and Engineering, Liaoning Technical University, Fuxin 123000, China

Density Functional Theory (DFT) Calculations

First-principles calculations with projected augmented wave (PAW) pseudopotential formalism were performed within the Perdew-Burke-Ernzerhof (PBE) exchange-correlation functional form of generalized gradient approximation (GGA) method as implemented in Vienna *Ab-initio* Simulation Package (VASP) software.[1-3] The electronic valence configurations for Pb and Te atoms were $5d^{10}6s^{2}6p^{2}$ and $5s^{2}5p^{4}$, respectively. The wave functions were adopted in plane wave basis with the kinetic energy cut-off of 500 eV. Due to the presence of heavy Pb element, the spin-orbital coupling effect (SOC)[4] was also separately considered in our calculations, which would affect the bandgaps and the positions of the band edges. The calculated lattice constant and formation enthalpy of 6.56 Å and -0.65 eV/atom were in accordance well with some previous works.[5-6] For conduct defect calculations, a 3×3×3 supercell containing 216 atoms for the NaCl structure was performed in this work, and the Monkhorst-Pack *k*-meshes[7] of 3 × 3 × 3 was used by the conjugated gradient method to sample in the Brillouin Zone (BZ). The convergence criterions for the total energy and Hellmann-Feynman force were less than 10^{-4} eV and 10^{-2} eV/Å, respectively. The lattice constant and ion position of the perfect supercell were relaxed, while for the supercells containing defects, the cell volumes were kept constant and the ion positions were relaxed.

The stability of a defect was determined by its formation energy, which is defined as:[8]

$$\Delta H_{\mathrm{d,q}} = E_{\mathrm{d,q}} - E_{\mathrm{pure}} - \sum_i n_i (E_i - \mu_i) + q(E_{\mathrm{V}} + E_{\mathrm{F}} + \Delta V)$$

Where represents the formation energy of a defect (d) in charge state (q). $E_{\mathrm{d,q}}$ and are the total energy of the defect system and perfect supercell, respectively. n_{i} and are the number and total energy of the *i*-th type (host atoms or impurity atoms) added to (n_i > 0) or taken from (n_i < 0) the supercells in order to create the defect. μ_i refers the corresponding chemical potentials of these atoms, which usually depends on experiment conditions. For a maximally rich growth environment of an element *i*, $\mu_i = 0$. q is the corresponding charge value of a charged defect. E_{F} is the Fermi level with respect to E_V , which is the valence band maximum (VBM) of the perfect supercell. ΔV indicates the electrostatic potential difference between the perfect supercell and defective system, which is aligned with the corresponding VBM. The difference of the

average electrostatic potential with the atoms far away from the defect sites was adopted in this work.[Error! Reference source not found.]

Formula

The average PF_{ave} value is calculated by:

$$PF_{ave} = \frac{\int_{T_c}^{T_h} PF(T)dT}{T_h - T_c}$$

where T_c is the cold-end temperature, T_h is the hot-end temperature.

Figures

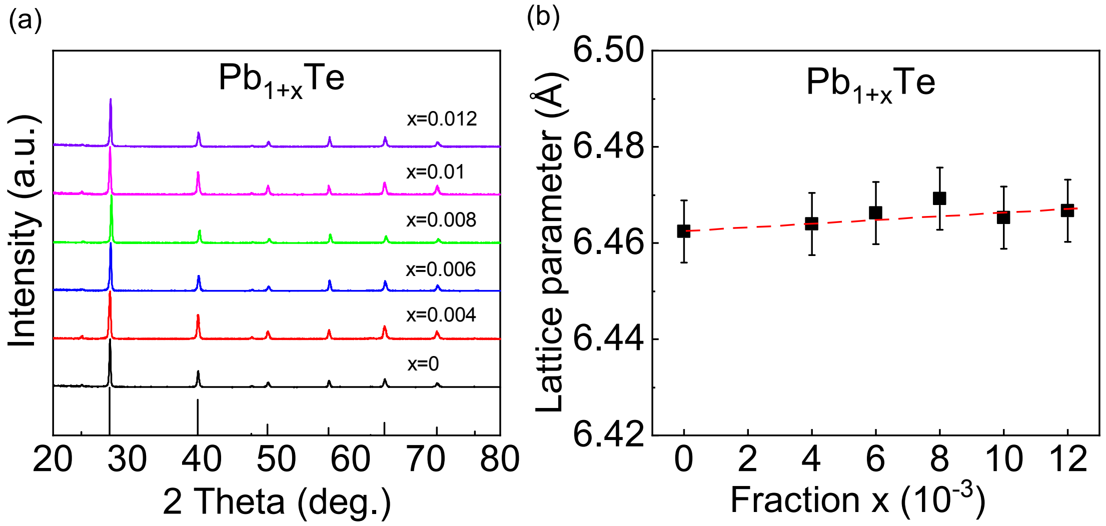

Figure S1. Phase identification of $Pb_{1+x}Te$ (x = 0, 0.004, 0.006, 0.008, 0.01, 0.0012): (a) powder XRD pattern; (b) lattice parameter.

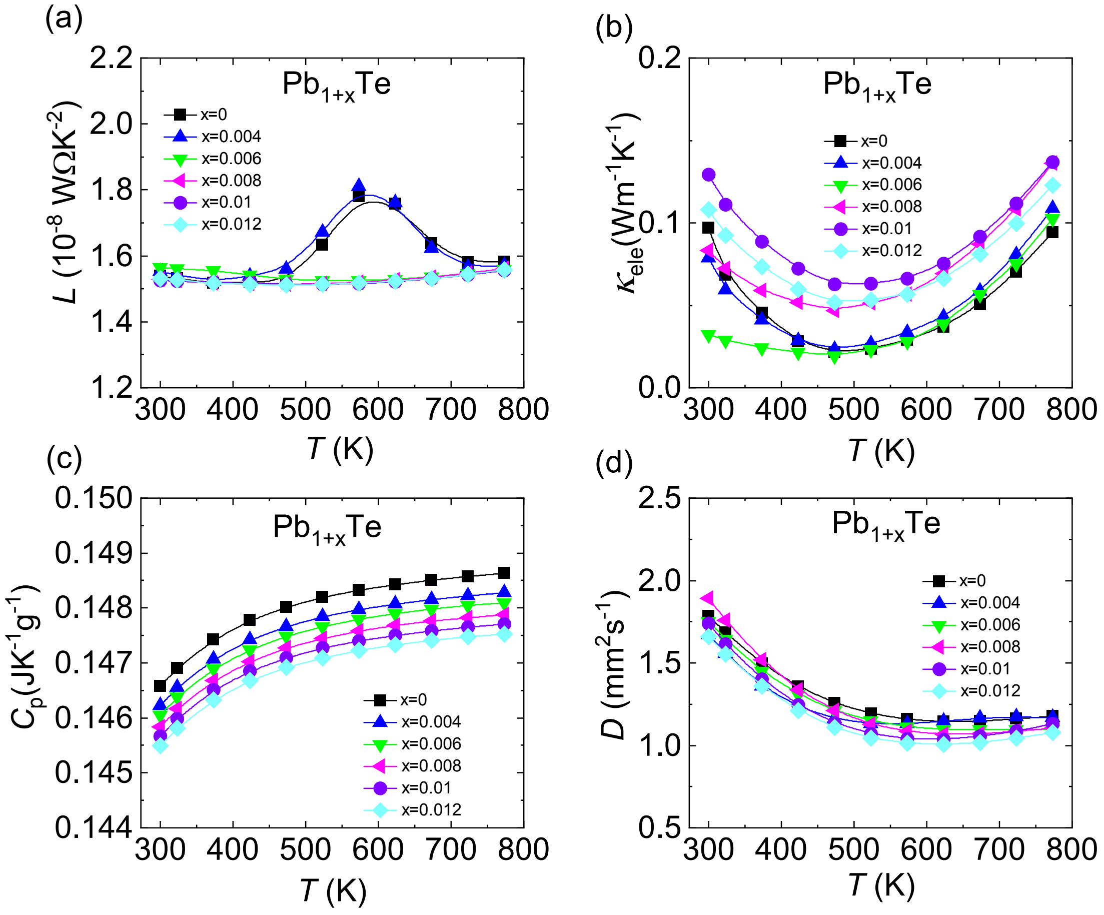

Figure S2. Thermoelectric transport properties as a function of temperature for $Pb_{1+x}Te$

(x = 0, 0.004, 0.006, 0.008, 0.01, 0.0012): (a) Lorenz number; (b) electronic thermal conductivity; (c) heat capacity; (d) thermal diffusivity.

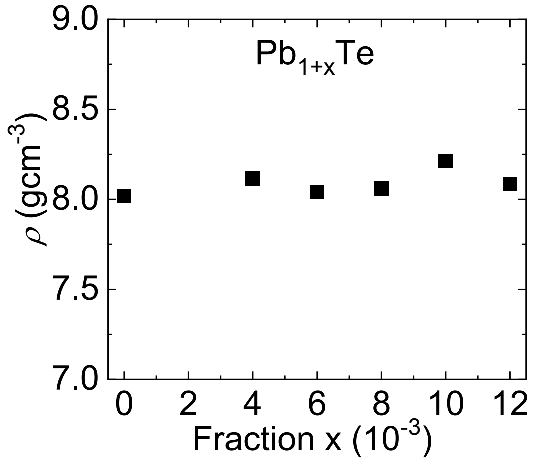

Figure S3. Sample density of $Pb_{1+x}Te$ (x = 0, 0.004, 0.006, 0.008, 0.01, 0.0012).

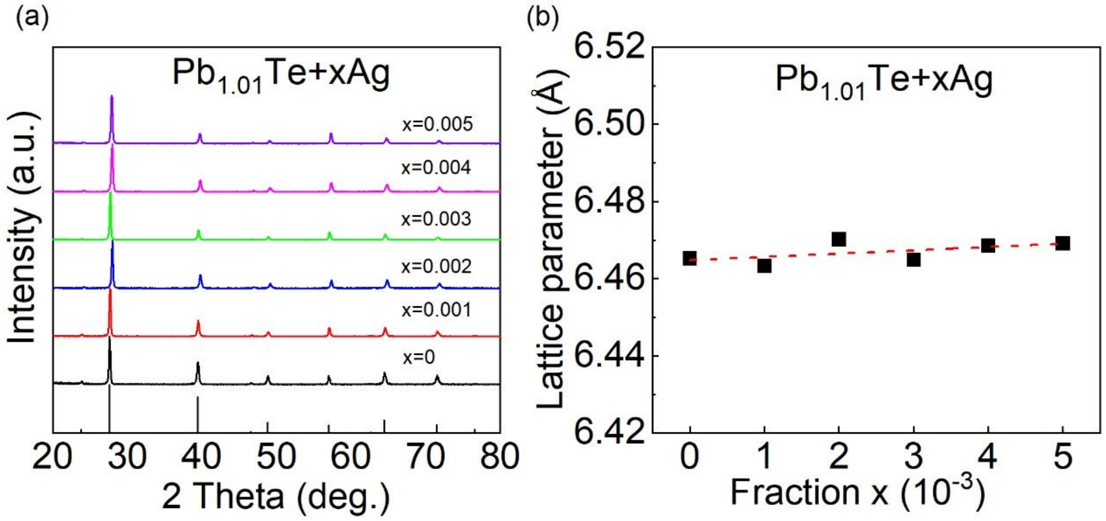

Figure S4. Phase identification of $Pb_{1.01}Te$+xAg (x = 0-0.005): (a) powder XRD pattern; (b) lattice parameter.

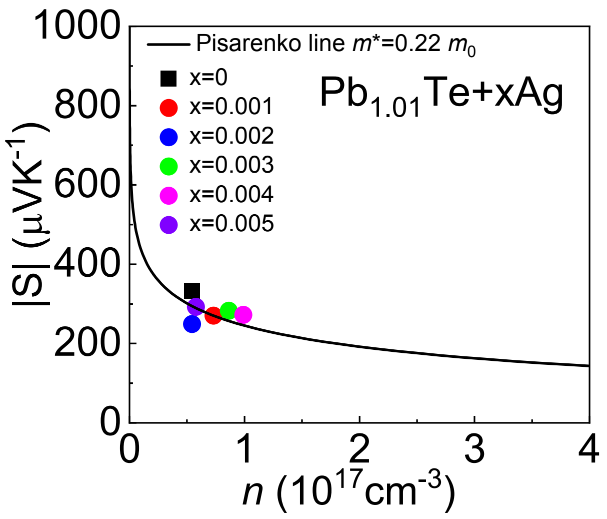

Figure S5. Room-temperature Pisarenko plots of $Pb_{1.01}Te$+xAg.

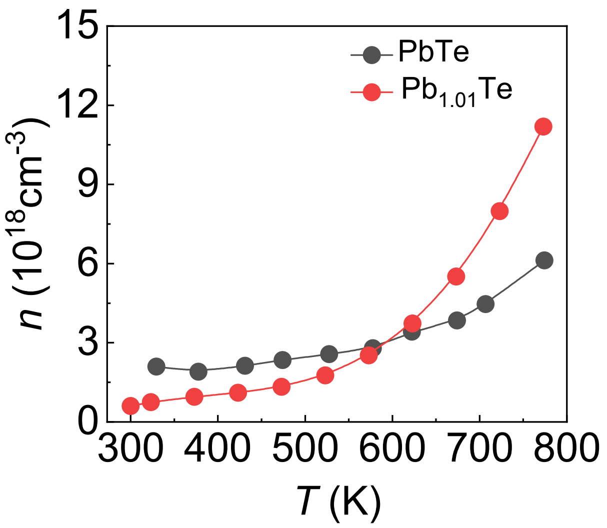

Figure S6. Carrier concentration of PbTe[9] and $Pb_{1.01}Te$ as a function of temperature.

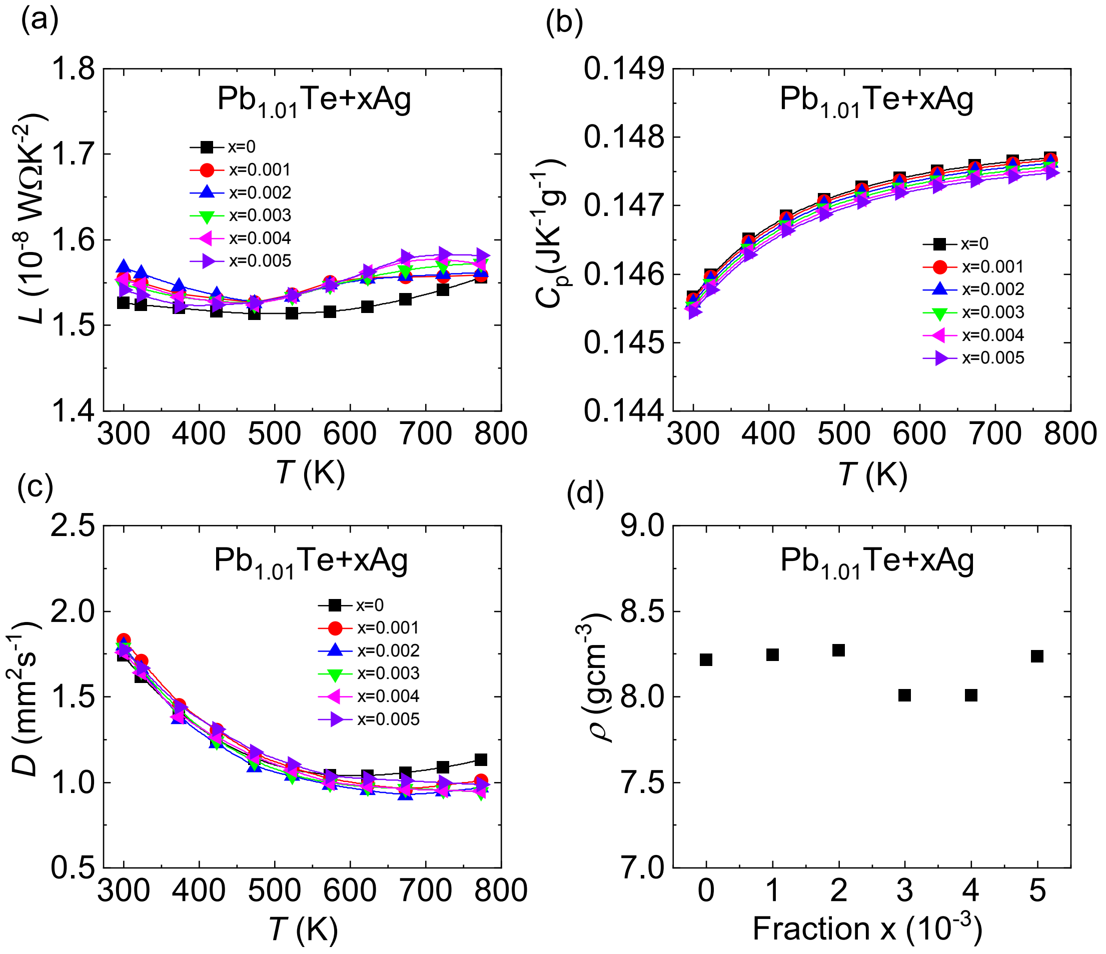

Figure S7. Thermoelectric transport properties as a function of temperature for $Pb_{1.01}Te$+xAg (x = 0-0.005): (a) Lorenz number; (b) electronic thermal conductivity; (c) thermal diffusivity; (d) sample density.

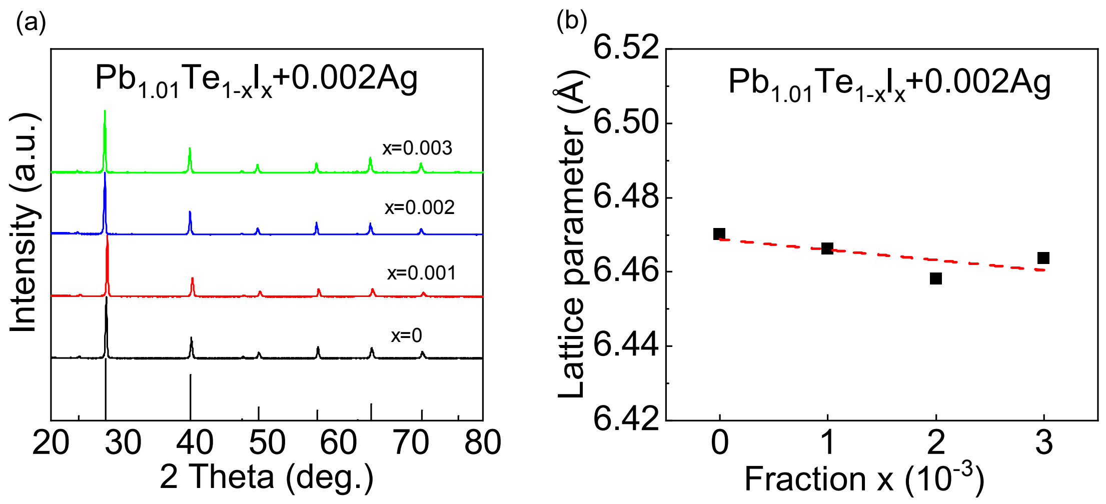

Figure S8. Phase identification of $Pb_{1.01}Te_{1-x}I_x$+0.002Ag (x = 0-0.003): (a) powder

XRD pattern; (b) lattice parameter.

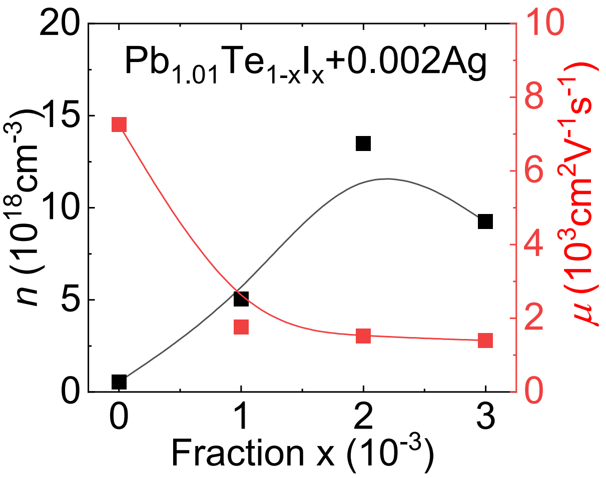

Figure S9. Room-temperature carrier density and carrier mobility as a function of Ag doping content.

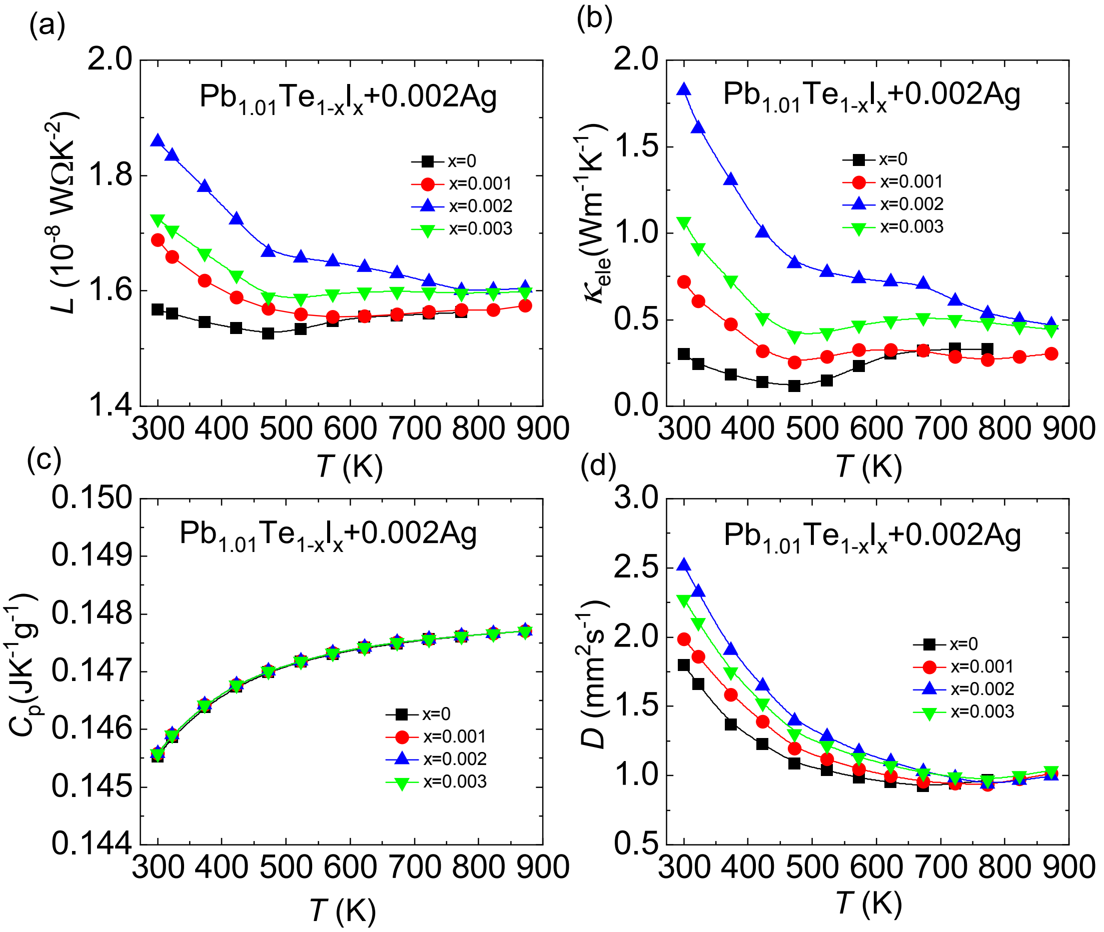

Figure S10. Thermoelectric transport properties as a function of temperature for $Pb_{1.01}Te_{1-x}I_x$+0.002Ag (x = 0-0.003): (a) Lorenz number; (b) electronic thermal

conductivity; (c) heat capacity; (d) thermal diffusivity.

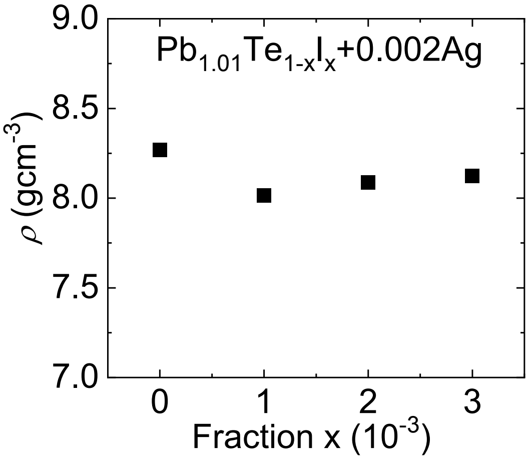

Figure S11. Sample density of $Pb_{1.01}Te_{1-x}I_x$+0.002Ag (x = 0-0.003).

REFERENCES

1. Hafner, J., Ab-Initio Simulations of Materials Using VASP: Density-Functional Theory and Beyond. *J. Comput. Chem.* **2008,** *29* (13), 2044-2078.

2. John P. Perdew, K. B., Matthias Ernzerhof, Generalized Gradient Approximation Made Simple. *Phys. Rev. Lett.* **1997,** *77* (18), 3865.

3. Blochl, P. E., Projector Augmented-Wave Method. *Phys. Rev. B* **1994,** *50* (24), 17953-17979.

4. Filatov, M.; Cremer, D., Calculation of Indirect Nuclear Spin-Spin Coupling Constants within the Regular Approximation for Relativistic Effects. *J. Chem. Phys.* **2004,** *120* (24), 11407-11422.

5. Goyal, A.; Gorai, P.; Toberer, E. S.; Stevanovic, V., First-Principles Calculation of Intrinsic Defect Chemistry and Self-Doping in PbTe. *Npj Comput. Mater.* **2017,** *3*, 51.

6. Huo, H. Y.; Wang, Y. X.; Xi, L. L.; Yang, J.; Zhang, W. Q., The Variation of Intrinsic Defects in XTe (X = Ge, Sn, and Pb) Induced by the Energy Positions of Valence Band Maxima. *J. Mater. Chem. C* **2021,** *9* (17), 5765-5770.

7. Monkhorst, H. J.; Pack, J. D., Special Points for Brillouin-Zone Integrations. *Phys. Rev. B* **1976,** *13* (12), 5188-5192.

8. Van de Walle, C. G.; Neugebauer, J., First-Principles Calculations for Defects and

Impurities: Applications to III-Nitrides. *J. Appl. Phys.* **2004,** *95* (8), 3851-3879.

9. You, L.; Zhang, J. Y.; Pan, S. S.; Jiang, Y.; Wang, K.; Yang, J.; Pei, Y. Z.; Zhu, Q.; Agne, M. T.; Snyder, G. J.; Ren, Z. F.; Zhang, W. Q.; Luo, J., Realization of Higher Thermoelectric Performance by Dynamic Doping of Copper in n-type PbTe. *Energy Environ. Sci.* **2019,** *12* (10), 3089-3098.
